# The Relationships Between Neighborhood Environment and Cognitive Function in Later Life

**DOI:** 10.1093/geroni/igaf122.2729

**Published:** 2025-12-31

**Authors:** Midori Takayama, Yoshiko Ishioka, Ikuko Sugawara

**Affiliations:** Keio University, Yokohama, Kanagawa, Japan; O.P. Jindal Global University, Sonipat, Haryana, India; Musashino University, Nishitokyo, Tokyo, Japan

## Abstract

Although the role of the neighborhood environment in health is well documented, little is known about how the neighborhood affects cognitive function. This study aimed to examine how physical and social neighborhood environments affect cognitive function in later life. Furthermore, we examined the interaction effect of physical function and economic status on the relationship between the neighborhood environment and cognition. Data were obtained from a locally representative three-wave longitudinal study of older Japanese aged 74–86 years (N = 1064). We used subjective measures of two physical environmental factors (public facilities, such as community centers, and accessibility) and two social environmental factors (availability of social participation programs and social inclusion). MMSE was used to assess cognitive functioning. The results of multilevel analyses showed that the availability of social participation programs was positively associated with cognitive function and that the degree of social inclusion was negatively associated with cognitive function. Moreover, physical functioning had an interaction effect on the relationship between social inclusion and cognitive functioning. Among older adults with lower physical function, cognitive function was lower in those living in more socially inclusive neighborhoods than in those living in less socially inclusive neighborhoods. These results suggest that creating opportunities for social participation in neighborhoods contributes to maintaining cognitive function, whereas fostering social inclusiveness in neighborhoods contributes to older adults’ continued aging in place, even after their cognitive and physical function declines. These results reinforce the need for neighborhood-level interventions to maintain cognition in later life and facilitate aging in place despite cognitive decline.

